# Increased Expression of SHMT2 Is Associated With Poor Prognosis and Advanced Pathological Grade in Oral Squamous Cell Carcinoma

**DOI:** 10.3389/fonc.2020.588530

**Published:** 2020-10-09

**Authors:** Zhi-Zhong Wu, Shuo Wang, Qi-Chao Yang, Xiao-Long Wang, Lei-Lei Yang, Bing Liu, Zhi-Jun Sun

**Affiliations:** ^1^The State Key Laboratory Breeding Base of Basic Science of Stomatology (Hubei-MOST), Key Laboratory of Oral Biomedicine Ministry of Education, School and Hospital of Stomatology, Wuhan University, Wuhan, China; ^2^Department of Oral Maxillofacial Head and Neck Oncology, School and Hospital of Stomatology, Wuhan University, Wuhan, China

**Keywords:** SHMT2, oral squamous cell carcinoma, prognosis, TCGA, tumor microenvironment

## Abstract

This study focused on the expression of mitochondrial serine hydroxymethyltransferase (SHMT2) in oral squamous cell carcinoma (OSCC) and its correlation with clinical traits and the prognosis of OSCC patients. Immunochemical staining and Western blotting were used to quantify the expression of SHMT2 and related immune markers in OSCC. Using OSCC microarrays and The Cancer Genome Atlas (TCGA) database, we evaluated the association between SHMT2 and various clinical traits. We found that increased expression of SHMT2 was detected in OSCC and correlated with advanced pathological grade and recurrence of OSCC. By a multivariate Cox proportional hazard model, high expression of SHMT2 was shown to indicate a negative prognosis. In addition, in the OSCC microenvironment, increasing the expression of SHMT2 was associated with high expression levels of programmed cell death-ligand 1 (PD-L1), CKLF-like MARVEL transmembrane domain containing 6 (CMTM6), V-type immunoglobulin domain-containing suppressor (VISTA), B7-H4, Slug, and CD317. In the future, more effort will be required to investigate the role of SHMT2 in the OSCC microenvironment.

## Introduction

Mainly originating from the oral cavity, oral squamous cell carcinoma (OSCC) is one of the most common malignant tumors of the head and neck (excluding non-melanoma skin cancer) (1, 2). Of note, OSCC results in negative consequences, as advanced OSCC has a poor 5-year-survival rate, impacts activities of daily living, and disfigures the appearance of patients after surgeries (1, 3). Though increasing numbers of OSCC patients have benefited from novel immunotherapies, such as immune checkpoint blockade (ICB), the OSCC microenvironment is complicated and heterogeneous and only approximately 20% of OSCC patients can undergo treatment successfully (4–6). The mechanism of the OSCC microenvironment is not yet clear, and previous studies have demonstrated that both Epstein–Barr virus (EBV) infection and human papilloma virus (HPV) infection independently act as biomarkers of prognosis in head and neck squamous cell carcinoma (HNSCC) (6, 7). However, EBV infection and HPV infection are external factors, and neither of them can reflect the changes in intracellular molecules related to OSCC. Hence, these facts motivated us to research the biomarkers that can indicate the prognosis and the alteration of intracellular molecules related to OSCC in the OSCC microenvironment (7, 8).

Recently, it has been reported that the tumor microenvironment under hypoxia promotes immune escape and hypoxia in the OSCC microenvironment, indicating poor prognosis and reflecting the involvement of the metabolic activity in mitochondria (9). Since the Warburg effect was discovered to be associated with tumor progression, increasing numbers of scholars have investigated energy metabolism in the tumor microenvironment (10). Coincidentally, mitochondrial serine hydroxymethyltransferase (SHMT2) is a vital enzyme involved in one-carbon unit metabolism that catalyzes the metabolism of serine into glycine in OSCC (11). Intriguingly, serine, as a substrate of SHMT2, is related to the Warburg effect by the active one-carbon unit metabolism (10). Furthermore, it has been reported that SHMT2 is highly expressed in glioma, intrahepatic cholangiocarcinoma, and hepatocellular carcinoma (12), reflecting that SHMT2 is partly involved in the process of tumorigenicity (10, 12, 13). It has been suggested that SHMT2 is downstream of signal transducer and activator of transcription 3 (STAT3) and plays a key role in the conversion of prostate cancer to a more aggressive phenotype (14, 15). However, it is not clear if SHMT2 is expressed in the OSCC microenvironment and whether SHMT2 is related to the prognosis of OSCC patients. In this study, we evaluate the expression levels of SHMT2 in both OSCC and oral normal mucosa and its influence on prognosis outcome. Furthermore, we related SHMT2 expression to case information of the OSCC patients we enrolled and analyzed the correlations between SHMT2 and clinical traits (16).

Immune checkpoints including programmed cell death-ligand 1 (PD-L1), V-type immunoglobulin domain-containing suppressor (VISTA), and B7-H4 have been demonstrated to be associated with the OSCC microenvironment, and ICB has become a novel immunotherapy to overcome cancer (16–19). The epithelial–mesenchymal transition (EMT) is an oncogenicity mechanism in OSCC, and Slug is one of the classical markers involved in EMT (20, 21). For the sake of investigating the correlations between SHMT2 and the OSCC microenvironment, we analyzed the association between SHMT2 expression and related markers in the OSCC microenvironment, including immune checkpoints and EMT markers, by OSCC microarrays.

## Materials and Methods

### Bioinformatics Analysis

A total of 307 OSCC cases are available from The Cancer Genome Atlas (TCGA) database^1^. Meanwhile, the normalized FPKM (fragments per kilobase million) values, as corresponding expression samples originated from RNA-sequencing and Gene Expression Quantification data, were acquired from TCGA Data Portal (22). We then utilized the expression data from 2,452 genes, including the *SHMT2* gene (Ensembl ID: ENSG00000182199), and corresponding clinical characteristics of OSCC patients to construct a weighted gene co-expression network analysis (WGCNA). We used the R package “WGCNA” (version 1.68), which possesses the function of module clustering and network analysis, to perform co-expression network analysis using R software (version 3.6.1). We first preprocessed the expression sample into a format suitable for network analysis, removed obvious outlier samples with excessive numbers of missing entries, and matched the trait samples to the expression samples (23). Second, using the methods of automatic network construction and module detection, we selected β = 4 as the soft thresholding power, where co-expression similarity was raised to calculate adjacency to meet the fitness of the scale-free topology index (roughly 0.90). Meanwhile, we chose a relatively large minimum module size of 30 and a medium sensitivity (deepSplit = 2) to perform cluster splitting. Subsequently, we visualized the results of clustering using the hierarchical clustering dendrogram constructed by the R package “WGCNA” (version 1.68) (23). Third, we correlated modules clustered with external characteristics and identified the association between them, defined as gene significance (GS). Further, for each module, we defined a quantitative measure of module membership (MM) as the correlation of the module eigengenes (MEs) and the gene expression profile, allowing us to quantify the similarity of all genes on the array to every module. By plotting a scatterplot of GS vs. MM, we conducted intramodular analysis to determine the correlation between MM and GS for the most positive traits. Finally, we conducted Gene Ontology (GO) and Kyoto Encyclopedia of Genes and Genomes (KEGG) analysis for the MEs using the R package “clusterProfiler” (version 3.12.0).

### Oral Squamous Cell Carcinoma Patients and Tissue Microarrays

Typical OSCC tissues and adjacent epithelial tissues, originated from OSCC patients and fixed by paraffin, were selected to punch cylindrical cores (1.5 mm) to construct OSCC and adjacent tissue microarrays. The corresponding case information of the OSCC patients has been reported as previously described (24). The OSCC and tissue microarrays consist of 176 primary OSCC samples, 25 recurrent OSCC samples, 68 metastatic lymph node samples of OSCC, 69 dysplasia tissue samples, and 42 adjacent normal oral mucosa samples. All the OSCC patients in this study signed an informed consent before surgery.

### Immunochemistry Staining and Immunochemistry Scoring Analysis, Hierarchical Clustering, and Visualization

An immunochemistry experiment was performed as previously described (25). After deparaffinization and dehydration by graded ethanol, the tissues on the microarrays were subjected to antigen retrieval with citric acid buffer (pH = 6.0) in a microwave. The tissues were incubated with 3% H_2_O_2_ and 10% normal goat serum at 37°C for 1 h in sequence. Next, we mixed the tissues of microarrays with the diluent antibody [SHMT2, Cell Signaling Technology (CST); CKLF-like MARVEL transmembrane domain containing 6 (CMTM6), Sigma-Aldrich; VISTA, CST; B7-H4, CST; PD-L1, CST; Slug, CST; CD317, Abcam] solution at 4°C in the refrigerator overnight. The next day, the tissues of the microarrays were mixed with the goat anti-rabbit IgG solution and avidin–biotin–peroxidase reagent solution at 37°C for 1 h in sequence. After staining with 3,3′-diaminobenzidine tetrachloride, the tissues were stained with hematoxylin again. To analyze the sample staining results, we utilized an Aperio ScanScope CS2 scanner (Vista, CA, United States) to scan the samples of microarrays and to quantify the histoscore at the area we chose from each microarray tissue using Aperio quantification software (Version 9.1) (26, 27). The detailed method for the analysis was as reported previously (25). Using Cluster 3.0, we performed hierarchical clustering analysis among SHMT2 and other correlative proteins (25). Java TreeView was applied to visualize the correlations described above (28).

### Human Oral Squamous Cell Carcinoma Specimens

Three pairs of OSCC samples and normal oral mucosa samples from three OSCC patients, who received treatment or surgery at the Hospital of Stomatology of Wuhan University during October 2019∼December 2019, were prepared for protein extraction. All patients were informed of and agreed with this study before the surgery. Additionally, the Medical Ethics Committee of the School and the Hospital of Stomatology of Wuhan University agreed with the study.

### Western Blotting

We performed Western blotting according to the established protocol (29). The protein samples extracted in the experiment mentioned above were first measured by a bicinchoninic acid assay (Beyotime Biotechnology, China) to detect the protein concentrations of the samples. We used 10% polyacrylamide gel (Servicebio, Wuhan, China) to conduct electrophoresis and transferred the protein (30 mg/lane) onto a polyvinylidene fluoride (PVDF) membrane. The proteins on the PVDF membrane were blocked in 5% defatted milk (Servicebio, Wuhan, China) for 1 h at room temperature. The PVDF membrane was then placed in dilute-antibody solution [SHMT2, CST, 1:1,000; glyceraldehyde-3-phosphate dehydrogenase (GAPDH), CST, 1:1,000] at 4°C in the refrigerator overnight. In the next morning, we took the PVDF membrane out of the solution, washed it with TBST solution three times, and placed it in 5% defatted milk with goat anti-rabbit IgG (HRP-label, Proteintech, Wuhan, China) at a dilution concentration of 1:10,000 at room temperature on a shaking table for 1 h. Finally, we utilized the WesternBright Sirius Chemiluminescent Detection Kit (Advansta, San Jose, CA, United States) to detect the membrane. The experiment was performed three times.

### Statistical Analysis

The analyses were carried out and visualized using GraphPad Prism (version 7.0) and Statistical Product and Service Solutions (SPSS, version 20.0). We used one-way ANOVA to conduct multiple group comparisons, and we used the *t*-test to conduct two-group comparisons. We arrayed the histoscore and the FPKM value in order of size and regarded the medial value of the histoscore and FPKM value as their median value. The best cutoff value is defined as the most significant cutoff value to separate two parts from a group based on the overall survival rate. Kaplan–Meier survival and multivariate analyses were conducted as described (25). For the correlation analysis of protein expression, we conducted a two-tailed Pearson statistical analysis.

## Results

### Overexpression of SHMT2 in Human Oral Squamous Cell Carcinoma

**FIGURE 1:**
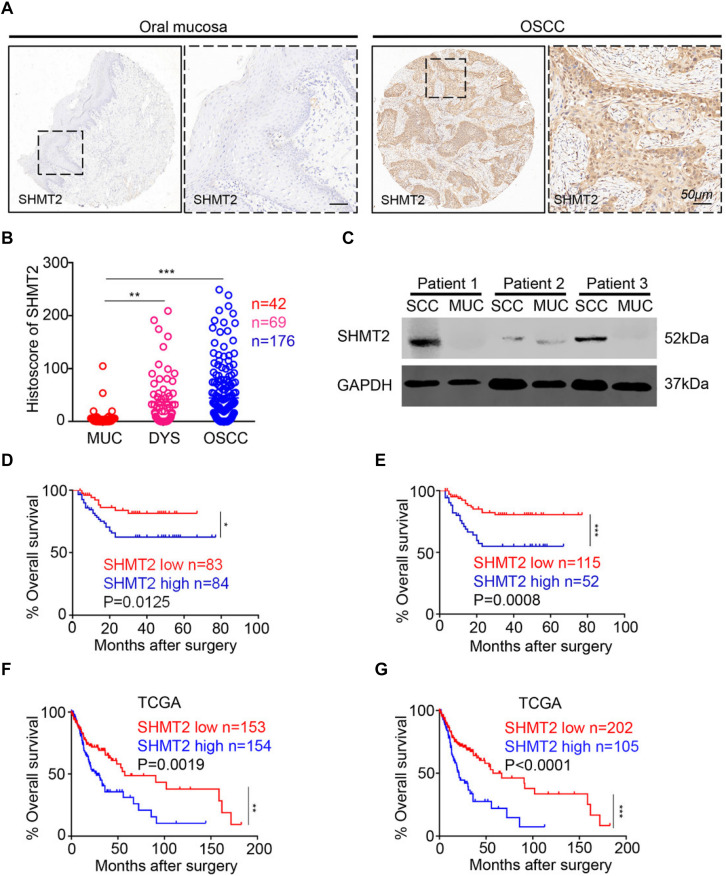
Overexpression of serine hydroxymethyltransferase (SHMT2) in primary oral squamous cell carcinoma (OSCC). **(A)** Representative immunohistochemical staining of SHMT2 in oral mucosa (left) and primary OSCC (right). The scale bar represents 50 μm. **(B)** Histoscores of SHMT2 as SHMT2 expression levels in OSCC (*n* = 176), dysplasia tissue (DYS, *n* = 69), and normal oral mucosa (MUC, *n* = 42). **(C)** The expression of SHMT2 in OSCC sample and oral mucosa sample of each OSCC patient (*n* = 3) was shown by Western blotting, and glyceraldehyde 3-phosphate dehydrogenase (GAPDH) was defined as a loading control. **(D,E)** Kaplan–Meier survival analysis of low and high expression of SHMT2 in OSCC based on microarrays]the median value was used for **(D)**, log-rank analysis; the best cutoff value was used for **(E)**, log-rank analysis]. **(F,G)** Kaplan–Meier survival analysis of low and high expression of SHMT2 in OSCC based on The Cancer Genome Atlas (TCGA) database [the median value was used for **(F)**, log-rank analysis; the best cutoff value was used for **(G)**, log-rank analysis]. Data are represented as the mean ± SEM and analyzed by one-way ANOVA with *post hoc* Tukey test or log-rank analysis. *P < 0.05; **P < 0.01; ***P < 0.001.

In this study, we utilized human OSCC tissue microarrays to investigate the expression of SHMT2 among human normal oral mucosa, dysplasia tissues, and OSCC by immunohistochemistry. As shown in Figure 1A, SHMT2, mostly expressed in cytoplasm, was stained in epithelial tissues and cancer cells and was rarely detected in stroma and immune cells. Meantime, lower expression of SHMT2 was detected in human normal oral mucosae compared with the expression of SHMT2 in dysplasia tissues (Figure 1B) and OSCC (Figure 1B), while there was no significant difference between dysplasia tissues and OSCC. In addition, we found that SHMT2 was indeed expressed at a high level in OSCC by comparison of the protein expression of the OSCC and normal oral mucosae of three OSCC patients (Figure 1C).

### Escalated Expression of SHMT2 Indicates a Negative Prognosis of Oral Squamous Cell Carcinoma Patients

To study the influence of the expression level of SHMT2 on the prognosis of OSCC patients, we performed Kaplan–Meier survival analysis on the data from the microarrays and TCGA database. Using the median value (histoscore = 21.57) and the best cutoff value (histoscore = 49.74) of microarrays as cutoff values, respectively, the results showed that the OSCC patients with a high SHMT2 expression had a poorer survival rate in comparison with those with a low SHMT2 expression (Figures 1D,E). Of note, the survival analysis based on the data from TCGA database demonstrated the same results: the high SHMT2 expression of OSCC patients is associated with a poorer survival outcome compared with low SHMT2 expression of OSCC patients when the median value (FPKM = 24.64) or best cutoff value (FPKM = 28.26) was used as a cutoff value (Figures 1F,G).

In addition, we put the clinical information for the OSCC patient cohort of microarrays, including gender, sex, history of drinking and smoking, pathological grade, node stage, tumor size and survival outcome, and expression of SHMT2, together to construct the multivariate Cox proportional hazard model. By multivariate analysis and using the best cutoff value, the results showed that escalated expression of SHMT2 was related to poor prognosis in OSCC (Table 1). Moreover, we utilized acquired data from TCGA database to reconstruct the multivariate Cox proportional hazard model, and the results of the multivariate analysis suggest that SHMT2 is a negative prognosis marker of OSCC patients (Tables 2, 3).

**TABLE 1:** Multivariate analysis for overall survival in primary OSCC patient cohort of microarrays (best cutoff value was used as a cutoff value).

**Parameters**	**HR (95% CI)**	***P*-value**
Gender	0.996 (0.380∼2.609)	0.993
Age	1.640 (0.783∼3.435)	0.190
Smoking	0.999 (0.416∼2.398)	0.998
Drinking	0.587 (0.243∼1.422)	0.238
Pathological grade		
I + II vs. III	1.275 (0.514∼3.164)	0.600
Node stage		
N1 vs. N0	0.914 (0.368∼2.272)	0.846
N2 vs. N0	2.490 (0.952∼6.513)	0.063
Tumor size		
T2 vs. T1	1.202 (0.393∼3.676)	0.747
T3 vs. T1	1.675 (0.496∼5.650)	0.406
T4 vs. T1	2.101 (0.473∼9.329)	0.329
SHMT2 expression	2.474 (1.214∼5.044)	0.013*

Cox proportional hazards regression model; HR, hazard ratio; 95% CI, 95% confidence interval; *P < 0.05. OCSS, oral squamous cell carcinoma; SHMT2, serine hydroxymethyltransferase.

**TABLE 2:** Multivariate analysis for overall survival in primary OSCC patient cohort of TCGA database (median value was used as a cutoff value).

**Parameters**	**HR (95% CI)**	***P*-value**
Gender	1.044 (0.680∼1.604)	0.844
Age	1.290 (0.819∼2.034)	0.272
Race	1.148 (0.774∼1.702)	0.492
**Pathological grade**		
II vs. I	1.308 (0.704∼2.433)	0.396
III vs. I	1.757 (0.913∼3.381)	0.091
**Node stage**		
N1 vs. N0	1.050 (0.579∼1.094)	0.873
N2 vs. N0	1.733 (0.864∼3.476)	0.122
N3 vs. N0	0.000 (0.000)	0.983
**Tumor size**		
T2 vs. T1	2.354 (0.271∼20.407)	0.437
T3 vs. T1	4.402 (0.520∼31.441)	0.182
T4 vs. T1	3.262 (0.416∼25.564)	0.260
**Stage**		
Stage 2 vs. Stage 1	0.735 (0.062∼8.784)	0.808
Stage 3 vs. Stage 1	0.554 (0.051∼6.011)	0.627
Stage 4 vs. Stage 1	0.439 (0.040∼4.882)	0.503
SHMT2 expression	1.845 (1.203∼2.828)	0.005*

Cox proportional hazards regression model; HR, hazard ratio; 95% CI, 95% confidence interval; *P < 0.05. OCSS, oral squamous cell carcinoma; SHMT2, serine hydroxymethyltransferase; TCGA, The Cancer Genome Atlas.

**TABLE 3:** Multivariate analysis for overall survival in primary OSCC patient cohort of TCGA database (best cutoff value was used as a cutoff value).

**Parameters**	**HR (95% CI)**	***P*-value**
Gender	0.958 (0.622∼1.478)	0.848
Age	1.193 (0.751∼1.895)	0.455
Race	1.099 (0.739∼1.635)	0.642
**Pathological grade**		
II vs. I	1.309 (0.699∼2.450)	0.400
III vs. I	1.611 (0.831∼3.123)	0.158
**Node stage**		
N1 vs. N0	1.016 (0.559∼1.849)	0.958
N2 vs. N0	1.484 (0.739∼2.980)	0.267
N3 vs. N0	0.000 (0.000)	0.983
**Tumor size**		
T2 vs. T1	2.538 (0.289∼22.254)	0.401
T3 vs. T1	5.024 (0.636∼39.678)	0.126
T4 vs. T1	3.169 (0.404∼24.829)	0.272
**Stage**		
Stage 2 vs. Stage 1	0.796 (0.066∼9.604)	0.858
Stage 3 vs. Stage 1	0.515 (0.047∼5.656)	0.587
Stage 4 vs. Stage 1	0.558 (0.050∼6.212)	0.635
SHMT2 expression	2.306 (1.502∼3.542)	0.000*

Cox proportional hazards regression model; HR, hazard ratio; 95% CI, 95% confidence interval; *P < 0.05. OSCC, oral squamous cell carcinoma; SHMT2, serine hydroxymethyltransferase; TCGA, The Cancer Genome Atlas.

### Expression Level of SHMT2 Is Associated With Pathological Grades and Recurrence of Oral Squamous Cell Carcinoma Among Oral Squamous Cell Carcinoma Patients

**FIGURE 2:**
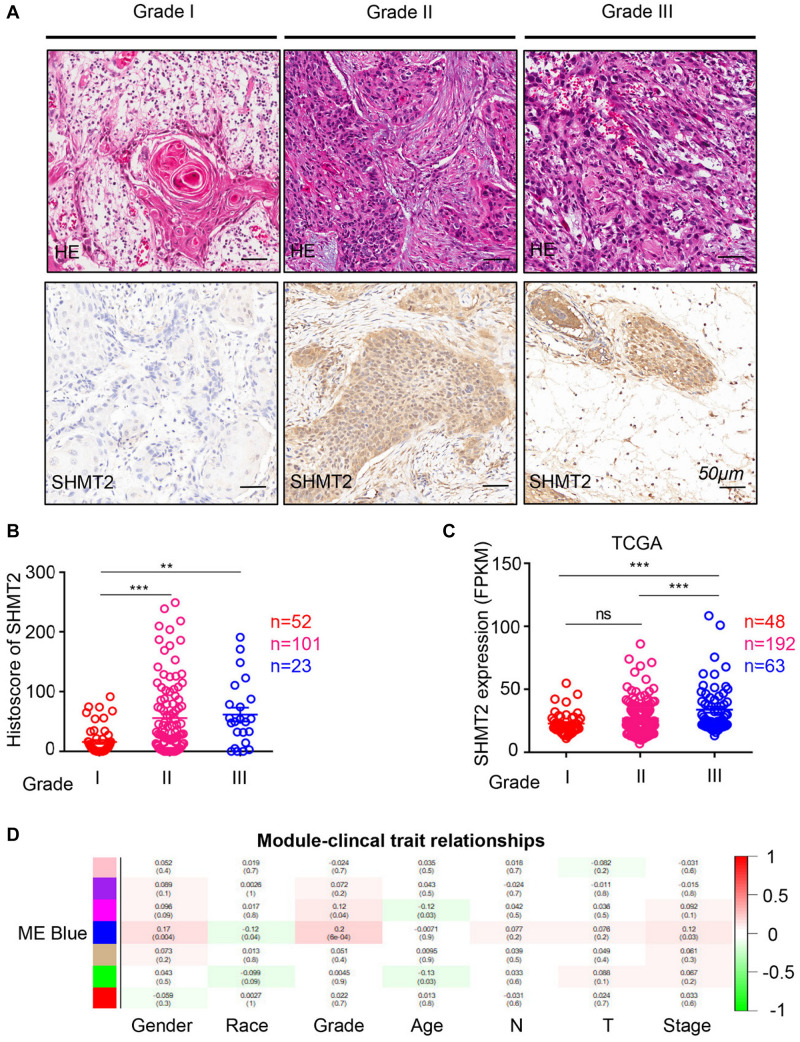
Increased expression of serine hydroxymethyltransferase (SHMT2) of oral squamous cell carcinoma (OSCC) patients with advanced pathological grade. **(A)** Representative hematoxylin–eosin (HE, Top) and immunohistochemical staining (bottom) of SHMT2 in Grade I tissue, Grade II tissue, and Grade III tissue. The scale bar represents 50 μm. **(B)** Histoscores of SHMT2 based on microarrays among Grade I tissues (*n* = 52), Grade II tissues (*n* = 101), and Grade III tissues (*n* = 23). **(C)** Expression of SHMT2 in OSCC based on The Cancer Genome Atlas (TCGA) database among Grade I tissues (*n* = 48), Grade II tissues (*n* = 192), and Grade III tissues (*n* = 63). **(D)** Correlations between module eigengenes (the *SHMT2* gene in the blue module) and clinical traits. Each unit of the table consists of the corresponding correlation coefficient and *P*-value. The color scale represents the strength of the correlation. Data are represented as the mean ± SEM and analyzed by one-way ANOVA with *post hoc* Tukey’s test. ns, no significance; **P < 0.01; ***P < 0.001.

**FIGURE 3:**
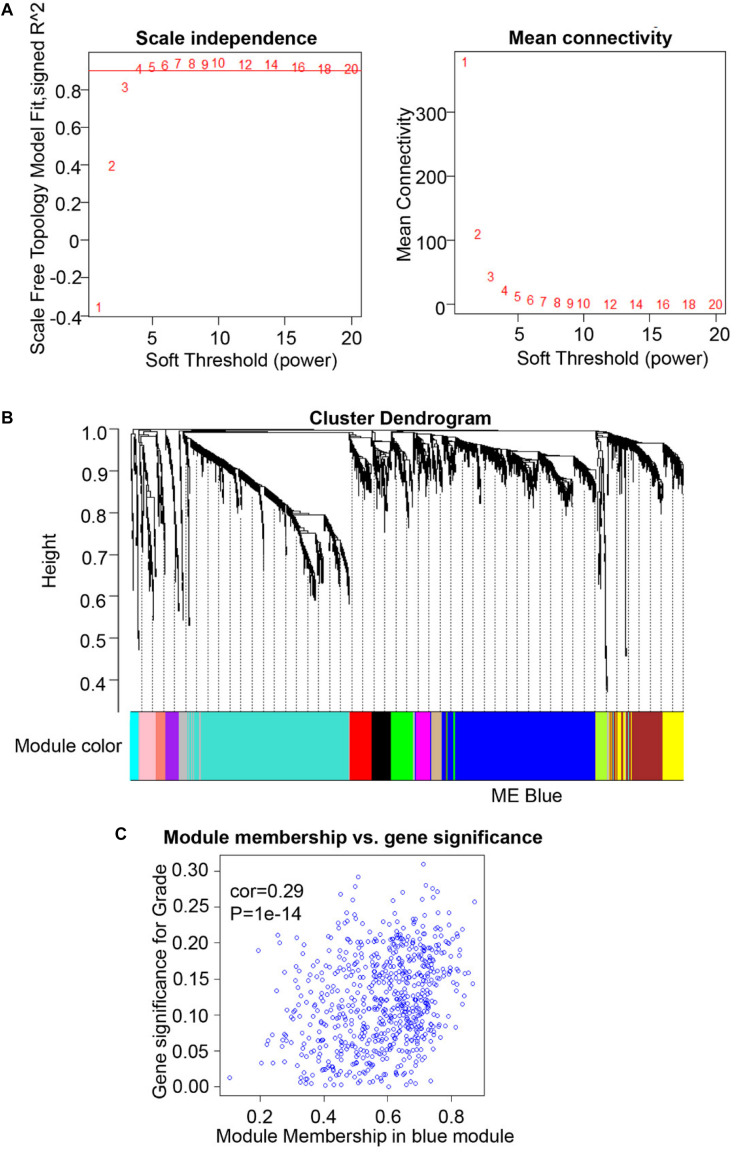
Weighted gene co-expression network analysis (WGCNA) of serine hydroxymethyltransferase (SHMT2)-related genes. **(A)** Analysis of scale-free topology for various soft-thresholding power. We selected β = 4 as the soft thresholding power. **(B)** Dendrogram of genes was clustered based on a dissimilarity measure (1-TOM). The genes were divided into several modules. Module Blue contained the *SHMT2* gene. **(C)** Scatter plot of gene significance for grade vs. module membership in the blue module (correlation coefficient = 0.29, *P* < 0.001).

To compare the SHMT2 expression of OSCC patients with different pathological grades based on microarrays and TCGA database, we used one-way ANOVA with a *post hoc* Tukey’s test and clearly observed that SHMT2 expression was related to different pathological grades (Figures 2A–C). According to the microarray data, the SHMT2 expression of OSCC patients with Grade I was significantly different from the expression in patients with Grade II and Grade III (Figure 2B), while the SHMT2 expression levels of OSCC patients with Grade I and Grade II were lower than those with Grade III according to TCGA database (Figure 2C). These results suggest that overexpression of SHMT2 of OSCC patients is related to advanced pathological grade. Furthermore, we utilized a transcription matrix of 2,452 genes, including the gene encoding SHMT2, acquired from TCGA database, to perform WGCNA (Figures 3A–C). Intriguingly, the outcome shows that Module Blue, which contains SHMT2, was strongly correlated with gender, tumor stage, and especially pathological grade (Figure 2D).

**FIGURE 4:**
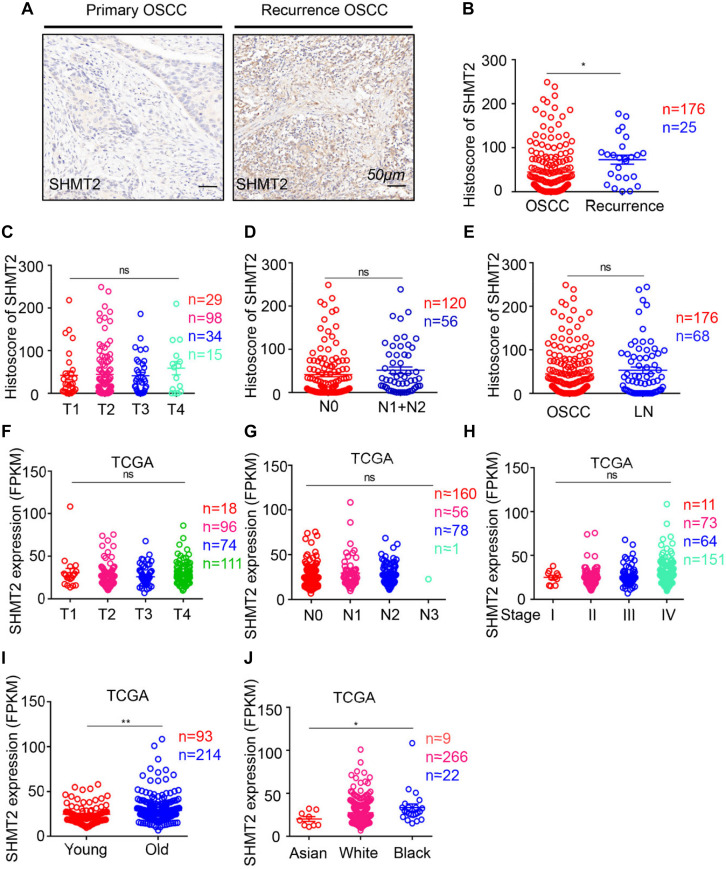
Escalated expression of serine hydroxymethyltransferase (SHMT2) of oral squamous cell carcinoma (OSCC) patients with recurrent OSCC. **(A)** Representative immunohistochemical staining of SHMT2 in primary OSCC (left) and recurrent OSCC (right). The scale bar represents 50 μm. **(B)** Histoscores of SHMT2 based on microarrays between primary OSCC (*n* = 176) and recurrent OSCC (*n* = 25). **(C–E)** Histoscores of SHMT2 based on microarrays in different tumor sizes, lymph node stages, and lymph node metastasis. **(F–J)** Expression of SHMT2 in OSCC based on The Cancer Genome Atlas (TCGA) database in different tumor sizes, lymph node stages, clinical stages, ages, and races. Data are represented as the mean ± SEM and analyzed by unpaired *t*-test or one-way ANOVA with *post hoc* Tukey test. ns, no significance; *P < 0.05; **P < 0.01.

Moreover, to determine the correlation between the SHMT2 and other clinical characteristics, we quantified the expression of SHMT2 by an immunochemistry scoring system and evaluated the differences in the target protein expression associated with different clinical traits. According to the microarray data, we identified a positive significance between increasing expression of SHMT2 and recurrent OSCC (Figures 4A,B), and there was no distinct difference in target protein expression among different tumor sizes (Figure 4C), lymph node stages (Figure 4D), and lymph node metastasis (Figure 4E). Coincidentally, an analysis based on the OSCC cases from TCGA database shows the similar consequence that there was no significance of SHMT2 expression among diverse tumor sizes (Figure 4F), lymph node stages (Figure 4G), and clinical stages (Figure 4H) but demonstrates that both age (Figure 4I) and race (Figure 4J) are correlated with SHMT2 expression to a certain extent.

### Gene Ontology and Kyoto Encyclopedia of Genes and Genomes Analysis

**FIGURE 5:**
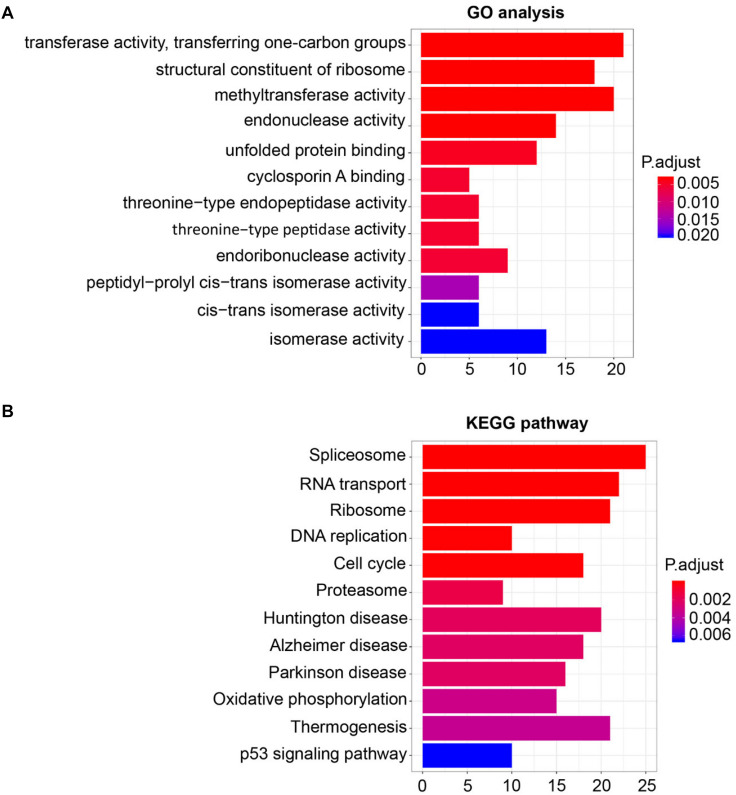
Gene Ontology (GO) and Kyoto Encyclopedia of Genes and Genomes (KEGG) pathway enrichment analysis for module eigengenes (MEs) of the blue module. **(A)** Plot of the enrichment GO terms GO analysis for MEs of the blue module (including the *SHMT2* gene). *Y*-axis represents the GO terms. *X*-axis represents the amount of MEs of the blue module enriched in the corresponding GO terms. **(B)** Plot of the enrichment KEGG pathway KEGG analysis for MEs of the blue module. *Y*-axis represents the KEGG pathways. *X*-axis represents the amount of MEs of the blue module enriched in the corresponding KEGG pathways. *P*-values were adjusted with the false discovery rate (FDR), and the adjusted *P*-value < 0.05 was the boundary to select GO terms and KEGG pathways.

For the sake of deeply detecting of the function of SHMT2, we utilized all genes of Module Blue to perform GO and KEGG analyses (30). As exhibited in Figure 5A, the most relative functions with the module genes, including SHMT2, include transferase activity transferring one-carbon groups, methyltransferase activity, and structural constituent of ribosome. Interestingly, by KEGG analysis, SHMT2-related genes were associated with some oncogenicity pathways, such as DNA replication, cell cycle, and especially the p53 signaling pathway (Figure 5B).

### Positive Correlations Among SHMT2 and PD-L1, CMTM6, VISTA, B7-H4, Slug, and CD317 in Oral Squamous Cell Carcinoma

**FIGURE 6:**
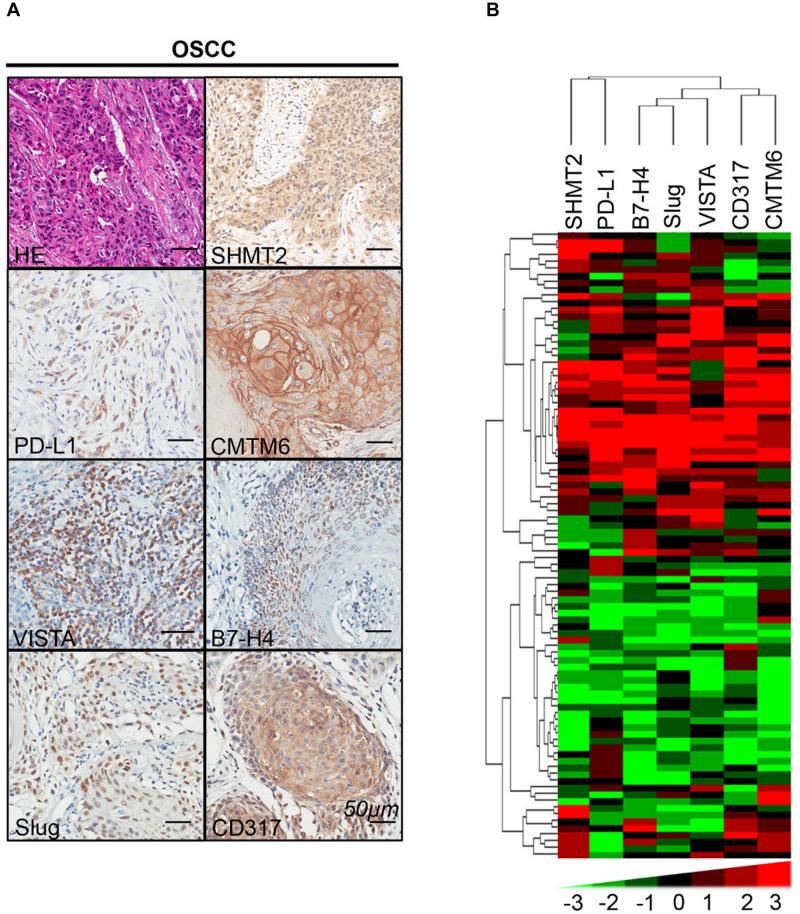
Correlations among serine hydroxymethyltransferase (SHMT2), programmed cell death-ligand 1 (PD-L1), CKLF-like MARVEL transmembrane domain containing 6 (CMTM6), V-type immunoglobulin domain-containing suppressor (VISTA), B7-H4, Slug, and CD317 in the oral squamous cell carcinoma (OSCC) microenvironment. **(A)** Representative hematoxylin–eosin and immunohistochemical staining of SHMT2, PD-L1, CMTM6, VISTA, B7-H4, Slug, and CD317 in OSCC. The scale bar represents 50 μm. **(B)** Strongly positive correlations among SHMT2, PD-L1, CMTM6, VISTA, B7-H4, Slug, and CD317 in OSCC shown by hierarchical clustering. The color scale represents the levels of histoscores.

**FIGURE 7:**
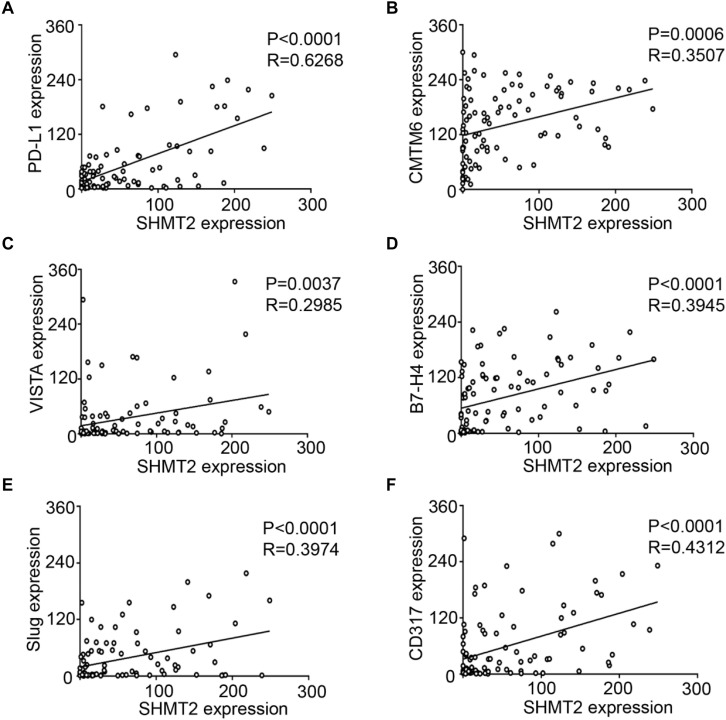
Statistical analysis of correlations among serine hydroxymethyltransferase (SHMT2), programmed cell death-ligand 1 (PD-L1), CKLF-like MARVEL transmembrane domain containing 6 (CMTM6), V-type immunoglobulin domain-containing suppressor (VISTA), B7-H4, Slug, and CD317 in the oral squamous cell carcinoma (OSCC) microenvironment. **(A–F)** The positive correlations among SHMT2 and PD-L1, CMTM6, VISTA, B7-H4, Slug, and CD317 in the OSCC microenvironment.

We found that SHMT2 expression was associated with related markers in the OSCC microenvironment (Figures 6A,B). The scatter plot shows that SHMT2 was strongly correlated with PD-L1 (Figure 7A), CMTM6 (Figure 7B), VISTA (Figure 7C), B7-H4 (Figure 7D), Slug (Figure 7E), and CD317 (Figure 7F) in OSCC patients. Of note, Slug is one of the protein markers involved in EMT, suggesting that SHMT2 may be involved in EMT or may be related to EMT in OSCC (20). Moreover, a high expression of SHMT2 is associated with increasing expression of some immune checkpoints, such as PD-L1 and VISTA, in OSCC, reflecting that SHMT2 is related to immune signaling in the OSCC microenvironment.

## Discussion

Being essential for a series of anabolic pathways, serine and glycine have been shown to be significant for the Warburg effect in cancer (31). Also, as an enzyme transferring serine and tetrahydrofolate into glycine and 5,10-methylenetetrahydrofolate, SHMT catalyzes the reaction that is required for *de novo* nucleotide biosynthesis and DNA methylation (31, 32). Owing to these characteristics, both SHMT isoforms including SHMT1 and SHMT2 play a key role in chemotherapeutic intervention (32). In the one hand, SHMT1, mostly located in the cytoplasm, regulates the partitioning of one-carbon units between deoxythymidine monophosphate (dTMP) and methionine (33). On the other hand, SHMT2, which exists in mitochondria, is more likely to give priority to take part in the synthesis of mitochondrial dTMP (32). In addition, SHMT2, as an enzyme involved in the metabolism of serine, is involved in tumorigenicity mediated *via* glycine (10, 34). However, the association between SHMT2 and clinical and pathological characteristics of OSCC has yet to be elucidated. In this article, we found an elevated expression of SHMT2 in OSCC compared to normal oral mucosa and a poor overall survival rate of OSCC patients with high SHMT2 expression. Of note, SHMT2 as an independent biomarker can indicate the prognosis of an OSCC patient cohort. More specifically, an escalated expression level of SHMT2 is related to a negative prognosis consequence in OSCC. In contrast, OSCC patients with a lower SHMT2 expression possess higher overall survival rates and better prognoses, similar with the results in breast cancer. These findings suggested that SHMT2, the high expression of which can slow down the process of glycolysis and augment the ratio of lactate and pyruvate, plays a vital role in the transition of PKM2, resulting in the glycolytic metabolic shift (10, 15, 34). It is known that one of the OSCC microenvironment features is hypoxia and that normal epithelial cells cannot adjust at poor oxygen pressure, so a high expression of SHMT2 may promote tumor cell survival for a long time in the OSCC microenvironment (9). Moreover, it is exactly that SHMT2 in mitochondria, not SHMT1 in the cytoplasm, was expressed highly in rapidly proliferating cancer cells (31, 35). When SHMT2 was inhibited, the proliferation of cancer cells was subsequently inhibited (31, 35).

In addition, an increasing expression of SHMT2 in OSCC is associated with advanced pathological grade and recurrent OSCC. In TCGA database, black people and aged individuals with OSCC are correlated with high expression levels of SHMT2 compared with Asians and young individuals, respectively. This discovery matched the previous finding that the role of the STAT3/SHMT2/PKM2 loop was found to convert prostate cancer to a more aggressive phenotype (34). Thus far, high expression levels of SHMT2 have been discovered in glioma and hepatocellular carcinoma (10, 12). SHMT2 expression was correlated with pathological grade, cell proliferation, migration, and EMT in hepatocellular carcinoma (12). Hence, gradually increasing levels of SHMT2 expression indicate a poor pathological state and one of the factors explaining why high SHMT2 expression represents a poor prognosis. These findings demonstrated that SHMT2 was correlated with tumors, particularly OSCC. Moreover, it has been proved that downregulation of SHMT2 can inhibit tumor occurrence rate and growth (34).

In our study, SHMT2 was found to be not only a biomarker of prognosis in OSCC but also a bridge correlating metabolic glycolysis with related markers of OSCC. In detail, SHMT2 was correlated with PD-L1, CMTM6, VISTA, B7-H4, Slug, and CD317 in the OSCC microenvironment. On the one hand, PD-L1, VISTA, and B7-H4 were correlated with the OSCC microenvironment, and their high expression indicates a suppressive status in OSCC (16–18). Additionally, CMTM6 is an important protein involved in the regulation of PD-L1, and CD317 is associated with B7-H4 and PD-L1 (36, 37). On the other hand, Slug is one of the classical markers related to EMT (20). The existence of EMT and immune checkpoints is significant for tumor escape (20, 38–40). Taken together, the correlations among SHMT2 and the related markers described above in OSCC may indicate a suppressive or impaired immune system preventing cancer cells from being attacked by T cells. Hence, the role and correlation of SHMT2 in the OSCC microenvironment possibly promote cancer cell growth in OSCC and lead to the poor prognosis of OSCC patients. Interestingly, SHMT2-related genes are associated with DNA replication, cell cycle, and the p53 signaling pathway of OSCC patients from TCGA database.

To conclude, SHMT2, as an independent marker indicating prognosis, was highly expressed in OSCC patients, and overexpression of SHMT2 was found in advanced OSCC and recurrent OSCC. Moreover, SHMT2 is involved in some processes of tumorigenesis and is related to PD-L1, CMTM6, VISTA, B7-H4, Slug, and CD317 expression in the tumor microenvironment of OSCC. We still require more research into SHMT2 as an enzyme involved in glycolysis to determine what role SHMT2 plays in the OSCC microenvironment and the specific mechanism between SHMT2 and the immune microenvironment in OSCC.

## Data Availability Statement

The raw data supporting the conclusion of this article will be made available by the authors, without undue reservation.

## Ethics Statement

The studies involving human participants were reviewed and approved by the School and Hospital of Stomatology Wuhan University Medical Ethics Committee. Written informed consent to participate in this study was provided by the participants’ legal guardian/next of kin. Written informed consent was obtained from the individual(s), and minor(s)’ legal guardian/next of kin, for the publication of any potentially identifiable images or data included in this article.

## Author Contributions

Z-ZW contributed to the conception, design, data acquisition, and analysis, and drafted and critically revised the manuscript. SW, Q-CY, X-LW, and L-LY contributed to data acquisition and critically revised the manuscript. BL and Z-JS contributed to conception, data analysis, and interpretation, and drafted and critically revised the manuscript. All authors gave final approval and agreed to be accountable for all aspects of the study.

## Conflict of Interest

The authors declare that the research was conducted in the absence of any commercial or financial relationships that could be construed as a potential conflict of interest.

## Footnotes

1 https://portal.gdc.cancer.gov/
